# FimC binds to the promoter region of *agn43* to modulate autoaggregation

**DOI:** 10.3389/fcimb.2025.1591206

**Published:** 2025-05-30

**Authors:** Zhihao Wang, Xiangpeng Niu, Ningyuan Zhong, Lanfang Kong, Saqib Nawaz, Haiyang Zhang, Wei Jiang, Yuanyuan Liu, Jian Tu, Xiangan Han

**Affiliations:** ^1^ Shanghai Veterinary Research Institute, The Chinese Academy of Agricultural Sciences (CAAS), Shanghai, China; ^2^ Anhui Province Key Laboratory of Veterinary Pathobiology and Disease Control, College of Animal Science and Technology, Anhui Agricultural University, Hefei, China; ^3^ Research and Development Department, Qingdao Orisess Biotechnology Co., Ltd, Qingdao, China

**Keywords:** avian pathogenic *E. coli* (APEC), type 1 fimbriae chaperone protein *fimC*, autoaggregation, DNA-binding, biofilm, c-di-GMP

## Abstract

**Background:**

Avian pathogenic *Escherichia coli* (APEC) infection causes high mortality in chicks and leads to significant economic losses in the poultry industry. During the initial infection, APEC colonizes host cells using type 1 fimbriae and subsequently forms biofilms, resulting in persistent and chronic infections. *fimC* is a chaperone protein associated with type 1 fimbriae and plays a crucial role in the assembly of fimbriae. However, its regulatory role in *agn43*-mediated autoaggregation remains unclear.

**Methods:**

By constructing *fimC* gene mutant strains, the autoaggregation, motility, biofilm formation, and the adhesion and invasion ability to HD-11 cells were examined. The transcriptome and the electrophoretic mobility shift assay (EMSA) were used to screen and verify the regulation of *fimC* on downstream genes.

**Results:**

The results demonstrated that the lack of *fimC*, but not fimbriae, significantly increased autoaggregation (*p* < 0.001) while promoting the transcription of *agn43* (*p* < 0.01). Transcriptomic analysis showed that the deletion of *fimC* caused significant changes in the gene transcription levels in a variety of pathways, such as flagellar synthesis, biofilm formation, quorum sensing, and bis-(3′-5′)-cyclic diguanylic acid (c-di-GMP) metabolism. Further investigation revealed that *fimC* directly interacted with the promoter region of *agn43* and inhibited its transcription. In addition, both *fimC* and *agn43* had regulatory effects on biofilm formation, motility, adhesion, and invasion.

**Conclusion:**

This study demonstrated that *fimC* acts as an atypical DNA-binding protein to regulate the transcription of *agn43*. It also highlights the importance of *fimC* in the biofilm formation and adhesion ability of APEC, which provides new insights into the functions of the fimbrial chaperone protein *FimC*.

## Graphical Abstract

The autoaggregation of *fimC* mutan strain was significantly decreased. However, deletion of *fimC* and *agn43* resulted in significant restored of autoaggregation. Mechanically, *fimC* binds to the *agn43* promoter region and regulates its transcription. In addition, *fimC* and *agn43* affect the biological characteristics of APEC such as biofilm and adhesion.

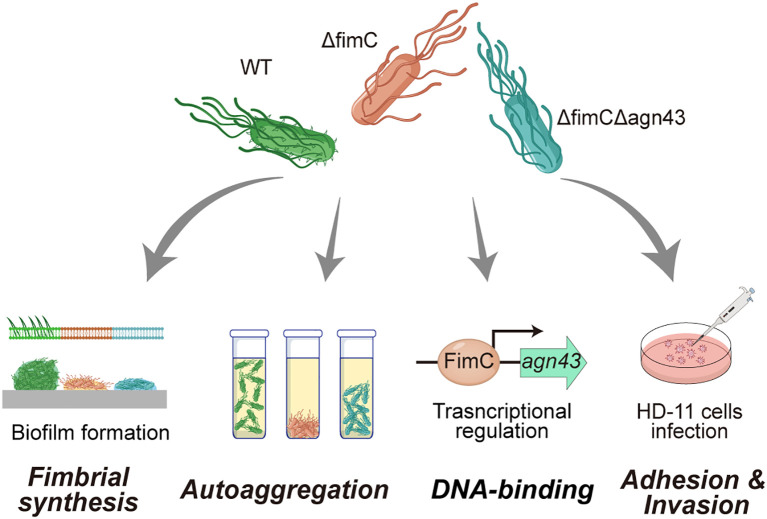

## 1 Introduction

Pathogenic *Escherichia coli* is a common pathogen that is typically divided into two major categories: intestinal pathogenic *E. coli* (InPEC) and extraintestinal pathogenic *E. coli* (ExPEC). Avian pathogenic *E. coli* (APEC) belongs to the ExPEC category. This disease has caused significant economic losses to the poultry industry due to increased mortality and decreased productivity (Barbieri et al., 2021; Li et al., 2016). APEC is also considered a foodborne pathogen associated with human urinary tract infections and is regarded as a potential reservoir for the transfer of virulence and antimicrobial resistance genes to human ExPEC strains, including those causing urinary tract infections (uropathogenic *E. coli*, UPEC) (Apostolakos et al., 2021; Kathayat et al., 2021).

APEC primarily induces avian diseases through its virulence factors, which include flagella, adhesins, autotransporters, iron acquisition systems, adhesins and invasins, and toxins (Nawaz et al., 2024). Adhesins, as components of the bacterial cell surface, promote adhesion to other cells or substances, typically within their host environment. During infection, adhesins initially contact host cells and trigger signaling pathways by recognizing specific receptors on the cell surface (Adlerberth et al., 1998; Arciola et al., 2015; Blumer et al., 2005; Hetta et al., 2021; Hoiby et al., 2010). This interaction is crucial for achieving colonization and the subsequent invasion.

Type 1 fimbriae play a vital role in the attachment to host cells and the initiation of biofilm formation, which are important processes in bacterial infections (Costa et al., 2015). These hair-like appendages are present on the outer membrane of APEC and specifically bind to mannose-containing residues. The *fim* gene cluster of the APEC strain includes *fimA*, *fimI*, *fimC*, *fimD*, *fimF*, *fimG*, and *fimH* (Spaulding et al., 2017). Under the guidance of the chaperone protein *fimC* and the usher protein FimD, these fimbrial subunits are assembled and fixed on the outer membrane. The *fimA* gene encodes the major subunit of type 1 fimbriae, while the *fimH* gene encodes the adhesin subunit that mediates the binding to α-d-mannosylated receptors, promoting bacterial adhesion to host epithelial cells (Pere et al., 1987). As a chaperone protein, *fimC* is crucial for the assembly of type 1 fimbriae (Jones et al., 1993). It aids in transporting the fimbrial subunit proteins to the periplasmic and, subsequently, to the outer membrane (Schembri and Klemm, 2001).

Autotransporters are a class of outer membrane proteins in *E. coli* that play a vital role in adhesion, autoaggregation, and biofilm formation. Antigen 43 (*agn43*) is a typical autotransporter in *E. coli* that promotes cell aggregation and enhances biofilm formation. Moreover, *agn43* contributes to bacterial survival within macrophages and sustains persistent urinary tract infections in the colorectal region (Court et al., 2003; Hasman et al., 2000; Korea et al., 2011). This multifunctionality underscores the importance of autotransporters in the pathogenesis and adaptability of *E. coli*. In addition, the AIDA (adhesin involved in diffuse adherence) and TibA (autotransporter adhesin/invasin) proteins appear to belong to an *E. coli* autotransporter subfamily, which has shown ~25% amino acid homology (Henderson and Nataro, 2001). Although these proteins are also virulent factors of bacteria and are involved in biofilm formation, the mechanisms of autoagglutination and biofilm regulation in *E. coli* still need to be explored further.

In this study, we elaborated the effects of *fimC* and *agn43* on biological characteristics such as autoagglutination, biofilm formation, and adhesion and explored the regulatory relationship between *fimC* and *agn43*, providing a reference for the prevention and control of APEC.

## 2 Materials and methods

### 2.1 Strains and plasmids, medium, and growth conditions

APEC81, a clinically isolated APEC O78 serotype strain, was preserved in our laboratory. pKD46, pCP20, and pKD3, which were used for gene knockout, were also preserved in our laboratory. The primers used in this study are shown in **Supplementary Table S1**. All strains were cultured at 37°C in Luria–Bertani (LB) broth. When necessary, the antibiotics ampicillin (Amp, 100 μg/ml), kanamycin (Kan, 50 μg/ml), streptomycin (Str, 50 μg/ml), and chloramphenicol (Cm, 35 μg/ml) were supplemented.

### 2.2 Construction of mutant and complementary strains

The Δ*fimC* strain was constructed based on the phage lambda-derived Red recombination system, with some modifications (Datsenko and Wanner, 2000). In brief, 400-bp DNA sequences upstream and downstream of the *fimC* gene from the APEC81 genome and the chloramphenicol resistance gene open reading frame (ORF) with the associated promoter and FRT (Flp recognition target) sites from pKD3 were connected using overlapping PCR. The PCR products were used to replace the 1,001-bp DNA within the *pdeN* gene with the Exo, Beta, and Gam proteins expressed by pKD46. The chloramphenicol resistance gene was eliminated by the Flp recombinase expressed by pCP20. The recombinant plasmid pSTV28–*fimC* was electro-transformed into the Δ*fimC* strain to construct the complementary strain Δ*fimC*–C*fimC*. The rest of the single gene mutant strains and the Δ*fimC*Δ*agn43* (based on the Δ*fimC* strain) strain were constructed as above.

### 2.3 Crystal violet staining

The biofilm formation ability was determined using crystal violet staining, with some modifications (Han et al., 2015). Briefly, 20 μl of each strain (OD_600_ = 1.0) was added in 96-well polystyrene plates (FCP962; Beyotime, Jiangsu, China) with 200 μl fresh LB broth and incubated at 37°C for 12 h. The cultures were gently discarded and the wells washed three times with 250 µl phosphate-buffered saline (PBS). After incubation at 60°C for 20 min, 250 μl crystal violet solution (1%) was added to the plates, which were then incubated at 37°C for 20 min, washed five times with 250 µl ddH_2_O, and air-dried completely at room temperature for 20 min. Images of crystal violet on the 96-well plates were captured. Crystal violet was resuspended *in situ* with 200 µl 95% ethanol and measured at an absorbance of 595 nm.

### 2.4 Transmission electron microscopy

Transmission electron microscopy (TEM) was performed as previously described (Primo et al., 2020). Each strain was cultured in LB at 37°C (OD_600_ = 1.0) and washed three times with PBS (pH 7.4). Of the cells, 10 μl was placed on a 200-mesh Formvar-coated copper mesh. After incubation at room temperature for 10 min, the samples were stained with 2% uranyl acetate aqueous solution for 30 s. After drying, the copper mesh was gently placed into a transmission microscope (HitachiHT-7700), as required, and the cell morphology determined.

### 2.5 Swimming motility assay

Determination of the swimming ability was performed with some modifications (Fredericks et al., 2006). In brief, using Petri dishes with semi-solid tryptone agar plates (1% bacto-tryptone, 0.5% sodium chloride, and 0.2% agar), 5 μl of each strain (OD_600_ = 1.0) was added into the center of the dishes and incubated for 8–10 h at 37°C. Free-swimming cells were captured.

### 2.6 Autoaggregation assay

The autoaggregation assay was performed as previously described (Elhenawy et al., 2021). Briefly, the strains (OD_600_ = 1.0) were collected and washed with PBS three times, then resuspended in PBS at OD_600_ = 2.0. All strains were incubated in stasis at 37°C. The absorbance at 600 nm of the upper suspension liquid was assessed using the same time interval.

### 2.7 Adhesion and invasion assay

Chicken macrophage DF-1 cells were cultured in Dulbecco’s modified Eagle’s medium (DMEM) supplemented with 10% fetal bovine serum at 37°C with 5% CO_2_. For the adhesion and invasion assay (Hu et al., 2011; Han et al., 2014), each strain was infected with DF-1 for 2 h in 24-well plates (multiplicity of infection, MOI = 50), the cells were collected and lysed using 0.5% Triton X-100, and the number of adherent bacteria were counted using the dilution method. After infection for 2 h, uninfected bacteria were discarded and a fresh culture medium supplemented with 100 μg/ml gentamycin sulfate was added. After incubation for 1 h, the subsequent processes were similar to those previously described.

### 2.8 RNA extraction and quantitative RT-PCR

RNA extraction and quantitative RT-PCR were performed with some modifications (Han et al., 2015). In brief, 15 ml of each strain was statically cultured in LB broth at 37°C and harvested at OD_600_ = 1.0. RNA was extracted with TRIzol. RNA sequencing was performed in Sangon Biotech (Shanghai, China). For quantitative RT-PCR, 2 ml of each strain (OD_600_ = 1.0) was harvested and the RNA extracted using TRIzol. The cDNA was reverse-transcribed using a Reverse Transcription Kit (R333; Vazyme Biotechnology, Nanjing, China). Quantitative PCR (qPCR) primers were designed, which are shown in **Supplementary Table S1**. qPCR was performed with the SYBR qPCR Kit (Q711; Vazyme Biotechnology) in Applied Biosystems QuantStudio 5. The 2^−ΔΔCt^ method was used for the calculation of fold change.

### 2.9 Protein expression and purification

The *fimC* gene ORF was amplified and constructed in the pET-28a plasmid. pET28a-*fimC* was transformed into BL21(DE3). The expression and the purification of the *fimC* protein were performed as previously described. Briefly, the BL21(DE3) strain was cultured in LB broth at 25°C and supplemented with 50 μg/ml kanamycin. The strains (OD_600_ = 0.4) were induced with IPTG (final concentration, 0.5 mM) at 28°C for 18 h. Bacterial cells were harvested and resuspended in PBS and then ultrasonically disrupted at 15% power for 3 min. The soluble His-tagged *fimC* protein retained on the Ni-NTA column was then eluted with increasing concentrations of imidazole buffer. Enriched proteins were analyzed using SDS-PAGE and were concentrated with ultrafiltration.

### 2.10 Western blot analysis

The promoter DNA of the *agn43* gene (from −262 to −1 bp) was fused with the flag-tagged *AmCyan* ORF (cyan fluorescent protein from *Anemonia majano*) in the low-cpy plasmid pACYC184. The plasmids were transformed into wild type (WT), Δ*fimC*, or the Δ*fimC*–C*fimC* strain. pACYC184 containing the Flag-tagged *amCyan* ORF without fusing any promoters was used as a negative control. For the Western blot analysis, 10 ml of each strain (OD_600_ = 1.0) was harvested and resuspended in PBS and then ultrasonically disrupted at 15% power for 3 min. Soluble proteins were analyzed using SDS-PAGE. After transfer to a PVDF membrane (ISEQ00010; Merck Millipore, Darmstadt, Germany) with the Trans-Blot^®^ Turbo™ Transfer Starter System (1704155), the PVDF membrane was blocked with 5% skimmed milk. An anti-flag monoclonal antibody was used for analysis of the expression of AmCyan (30502ES60; YEASEN, Shanghai, China). An anti-DnaK polyclonal antibody was used as reference (PH3459S; Abmart, Berkeley Heights, NJ, USA).

### 2.11 Electrophoretic mobility shift assay

Electrophoretic mobility shift assay (EMSA) was performed according to the protocol in the LightShift^®^ Chemiluminescent EMSA Kit (no. 20148; Thermo, Waltham, MA, USA). Briefly, 2.5 μg *fimC* protein was incubated at 37°C for 20 min in 20 μl reaction buffer [containing 1× binding buffer, 1 μg Poly (dI-dC), 2.5% glycerol, 0.05% NP-40, 5 mM Mn^2+^, and 5 mM Zn^2+^]. Approximately 1 ng cy5.5-labeled *agn43* promoter DNA or 100 ng unlabeled *agn43* promoter DNA was added and incubated at 37°C for 20 min. All samples were analyzed by electrophoresis in 4% native TBE polyacrylamide gels at constant 100 V for 60 min. The gel was scanned using a laser imager (Odyssey, LI-COR, Lincoln, NE, USA) with focus at 1.5 mm.

### 2.12 Statistical analysis

Each experiment was repeated at least three times. Data are presented as the mean ± standard deviation (SD). Student’s *t*-tests were performed to compare two groups, while one-way ANOVA and Dunn’s *post-hoc* tests for statistical analysis were used for comparisons among multiple groups. *P*-values were calculated using GraphPad Prism software (version 9.0). A *p* < 0.05 was considered statistically significant (*0.01 < *p* < 0.05, **0.001 < *p* < 0.01, ***p < 0.001).

## 3 Results

### 3.1 *fimC* is necessary for the biosynthesis of fimbriae

*fimC* has been reported as a chaperone protein associated with type 1 fimbrial synthesis (Nishiyama et al., 2003). The *fimA*, *fimC*, and *fimD* mutant strains were constructed for the analysis of the biosynthesis of fimbriae. The results showed that the transcription levels of *fimA*, *fimC*, and *fimD* were significantly decreased in these mutant strains (*p* < 0.001) (**Supplementary Figure S1**), while the transcription level of *fimC* in Δ*fimC*–C*fimC* was significantly increased compared with the Δ*fimC* strain (*p* < 0.001). All results showed that *fimA*, *fimC*, and *fimD* cannot be detected at the transcription level in their mutant strains, indicating that mutant strains were successfully constructed.

**Figure 1:**
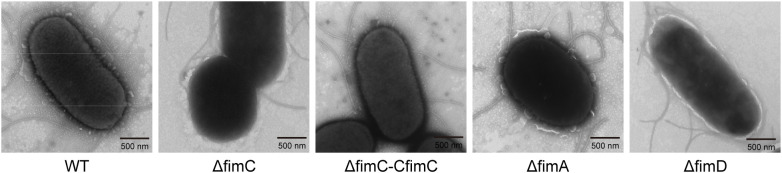
Observation of bacterial surface structure by transmission electron microscopy (TEM). 10 μL of ΔfimA, Δ*fimC*, Δ*fimC*-C*fimC* and Δ FimD mutan strains (OD600 = 1.0) were placed on a 200-mesh Formvar-coated copper mesh, samples (n=5) were stained with 2% uranyl acetate aqueous solution for 30 s. The copper mesh was gently placed into a transmission electron microscope (HitachiHT-7700) as required. Cell morphology were captured and processed by Fiji.

TEM showed that, in the WT strain, the fimbriae appeared thick and densely packed (**Figure 1**). In contrast, the Δ*fimC* strain displayed neither intact nor broken fimbriae. Furthermore, fimbriae appeared in the Δ*fimC*–C*fimC* strain. Moreover, *fimA* and *fimD* were deleted as controls, without showing either intact or broken fimbriae, which is consistent with previous reports (Giese et al., 2023; Nishiyama et al., 2008). The results indicate that the lack of *fimC* led to complete loss of the ability to synthesize type 1 fimbriae in APEC.

### 3.2 *fimC* represses the autoaggregation

**Figure 2:**
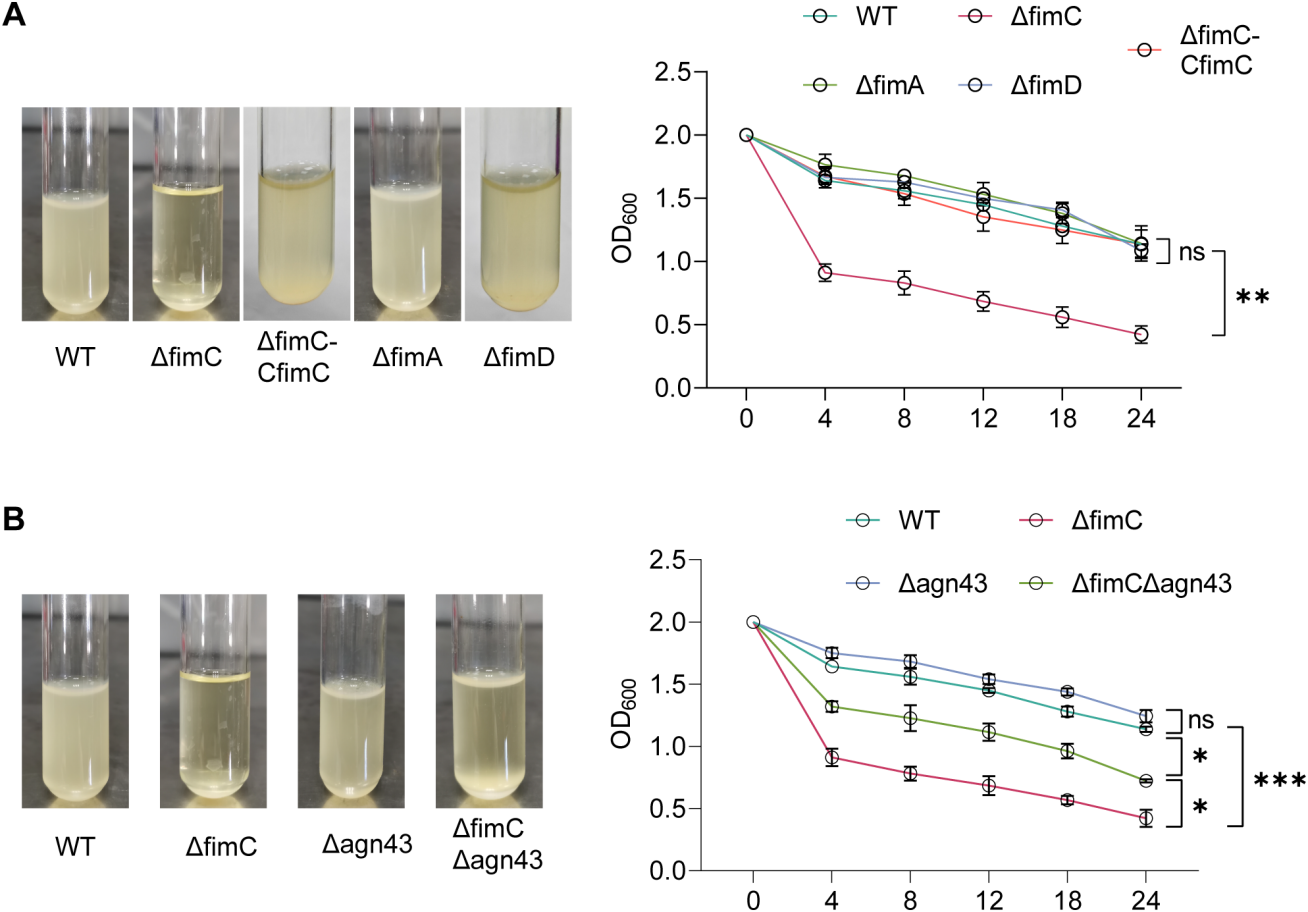
Detection of autoaggregation of APEC. **(A)** Detection of autoaggregation of type I fimbrial synthesis gene *fimC*, *fimA* and *fimD* mutant strain in test tubes (left). Detection of autoaggregation of type I fimbrial synthesis gene *fimC*, *fimA* and *fimD* mutant strain by absorbance 600 nm (right) (n=3). **(B)** Detection of autoaggregation of *agn43* mutant strain and Δ*fimC*Δ*agn43* mutant strain (n=3).

The *fim* gene cluster, as a type 1 fimbrial synthesis gene, determines the integrity of fimbrial synthesis. However, whether fimbriae affect autoaggregation remains unclear. Here, the autoaggregation results showed that, after incubating statically at 37°C for 24 h, the Δ*fimC* strain showed a significantly increased autoaggregation compared with the WT strain (*p* < 0.001) (**Figure 2A**). In addition, autoaggregation in the Δ*fimC*–C*fimC* strain was restored to WT levels (*p* > 0.05). Moreover, to further verify whether the increase in autoaggregation is caused by the inability to produce fimbriae, the effect of the *fimA* and *fimD* gene mutants on autoaggregation was established. The results showed that the autoaggregation of the ΔfimA and/or the ΔfimD strain remained no different from that of the WT strain (*p* > 0.05) (**Figure 2A**). These results indicate that, although either lacking *fimC* or *fimA/fimD* result in the inability to produce fimbriae, but only lacking *fimC* significantly increased autoaggregation.

### 3.3 Transcriptomic analysis reveals that deletion of *fimC* alters the transcription of multiple pathways

**Figure 3:**
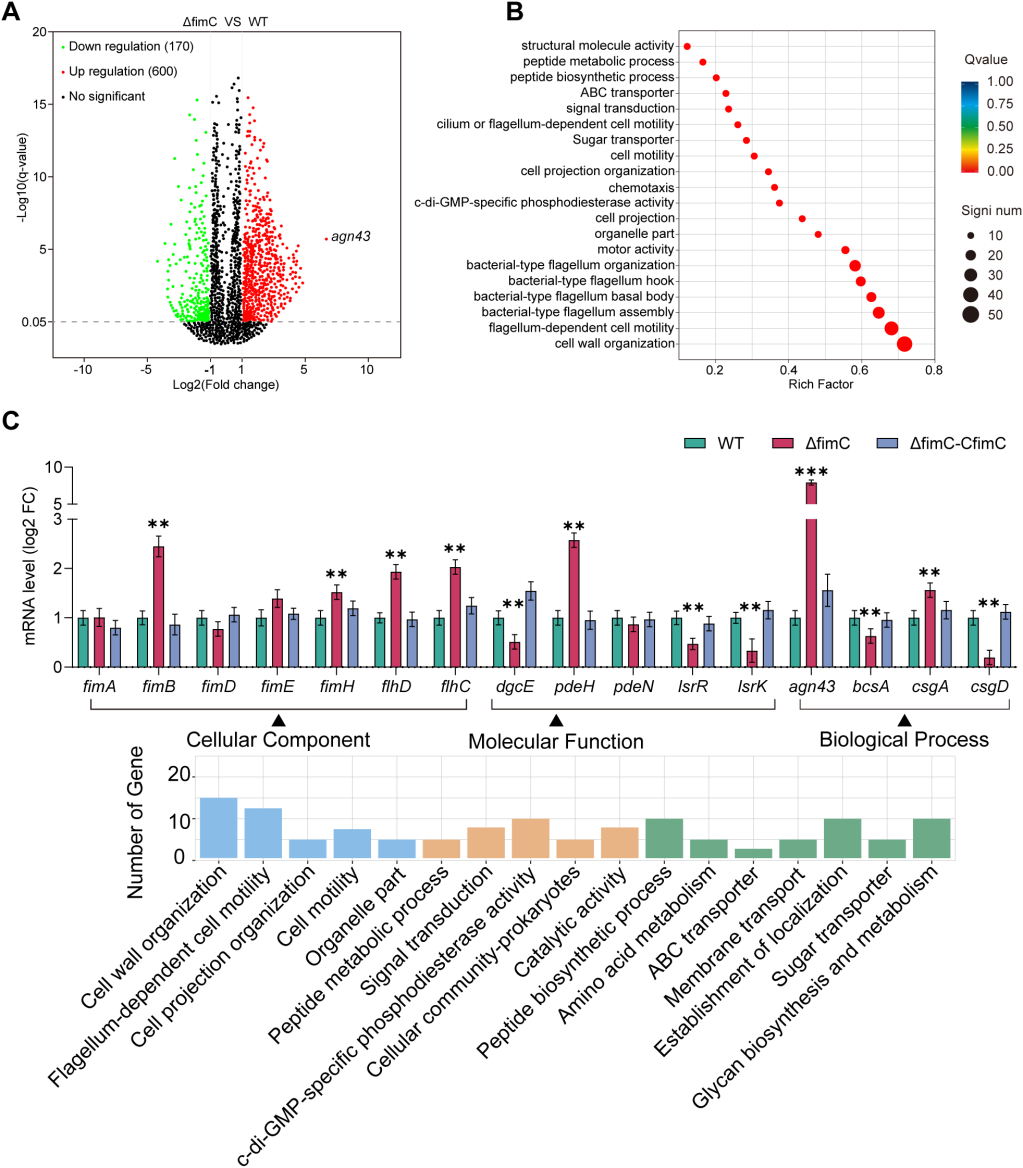
Transcriptomic and RT-qPCR analysis of genes with altered transcriptional level in Δ*fimC* strain. **(A)** Volcano plot shows the number of significantly differentially expressed genes in *fimC* mutant strain. **(B)** Enrichment analysis of clusters of orthologous groups of proteins (COG). **(C)** Detection of transcriptional level of genes classified into three categories by RT qPCR. In the transcriptome data, genes with altered transcriptional levels were grouped into three categories, Biological Process, Cellular Component and Molecular Function(top). Genes from these classifications were selected for qPCR to verify whether transcription levels of these genes changed after *fimC* deletion (bottom).

To explore the mechanism for the increased autoaggregation caused by the deletion of *fimC*, as well as other potential related clues, a transcriptomic analysis was performed. The results showed that the transcription levels of 600 genes were upregulated and that 170 genes were downregulated (**Figure 3A**). All genes were classified using gene function classification (Gene Ontology, GO) into three categories: biological process, cellular component, and molecular function (**Figure 3C**). GO enrichment analysis demonstrated the top 20 enriched pathways. Half of these pathways are involved in flagellum-dependent cell motility and cell adhesion (**Figure 3B**). In addition, the pathway of bis-(3′-5′)-cyclic diguanylic acid (c-di-GMP) metabolism was also enriched (**Figure 3C**). These results indicate that, although *fimC* is a chaperonin protein associated with type 1 fimbrial synthesis, its potential function may involve multiple regulatory pathways.

To further explore the function of *fimC* in cell adhesion, motility, and related potential regulatory functions, RT-qPCR was performed. All selected genes were classified according to the categories of transcriptome clustering. The results showed that the transcription level of the *fim* cluster genes significantly increased, particularly *fimB* (log_2_FC = 2.5) and *fimH* (log_2_FC = 1.5; *p* < 0.01). Furthermore, the transcription level of the flagellar primary transcription regulator *flhDC* was increased (log_2_FC = 2.0; *p* < 0.01). It has been reported that quorum sensing and c-di-GMP are important regulatory factors through small molecules associated with the regulation of cell autoaggregation, motility, and biofilm formation, among others. In this study, the transcription levels of the quorum sensing autoinducer-2 kinase *lsrK* (log_2_FC = −1.2) and the transcriptional regulator *lsrR* (log_2_FC = −1.5) were significantly decreased with the deletion of *fimC* (*p* < 0.01). The c-di-GMP metabolic genes such as *pdeH* were significantly increased (log_2_FC = 2.4, *p* < 0.01). Furthermore, the transcription level of the self-recognizing antigen 43 (*agn43*), a autotransporter, was significantly upregulated ~8.2-fold (*p* < 0.001) (**Figure 3C**). Taken together, these results showed that *fimC* is involved in multiple regulatory pathways in *E. coli*, demonstrating a complex regulatory network that has not yet been reported.

### 3.4 The autoaggregation regulated by *fimC* is mediated by *agn43*

It has been reported that *agn43*, as a self-recognizing antigen and autotransporter, mediates the autoaggregation and biofilm formation in *E. coli*; therefore, a Δ*agn43* strain was constructed (Sherlock et al., 2006). However, in this study, it was shown that the Δ*agn43* strain did not significantly influence the autoaggregation compared with the WT strain (*p* > 0.05) (**Figure 2B**). These data suggest that species diversity and gene compensatory effects may be the reasons for *agn43* deletion not affecting autoagglutination.

However, to explore whether there is a correlation between *fimC* and *agn43*, a Δ*fimC*Δ*agn43* mutant strain was constructed based on Δ*fimC*. The results showed that, although the autoaggregation of Δ*fimC*Δ*agn43* increased compared with WT (*p* < 0.05), the autoaggregation of the Δ*fimC*Δ*agn43* strain was significantly decreased compared with that of Δ*fimC* (*p* < 0.05). Taken together, these results indicate that *fimC* represses the autoagglutination and is mediated by *agn43*.

### 3.5 *fimC* inhibits the transcription of *agn43*

**Figure 4:**
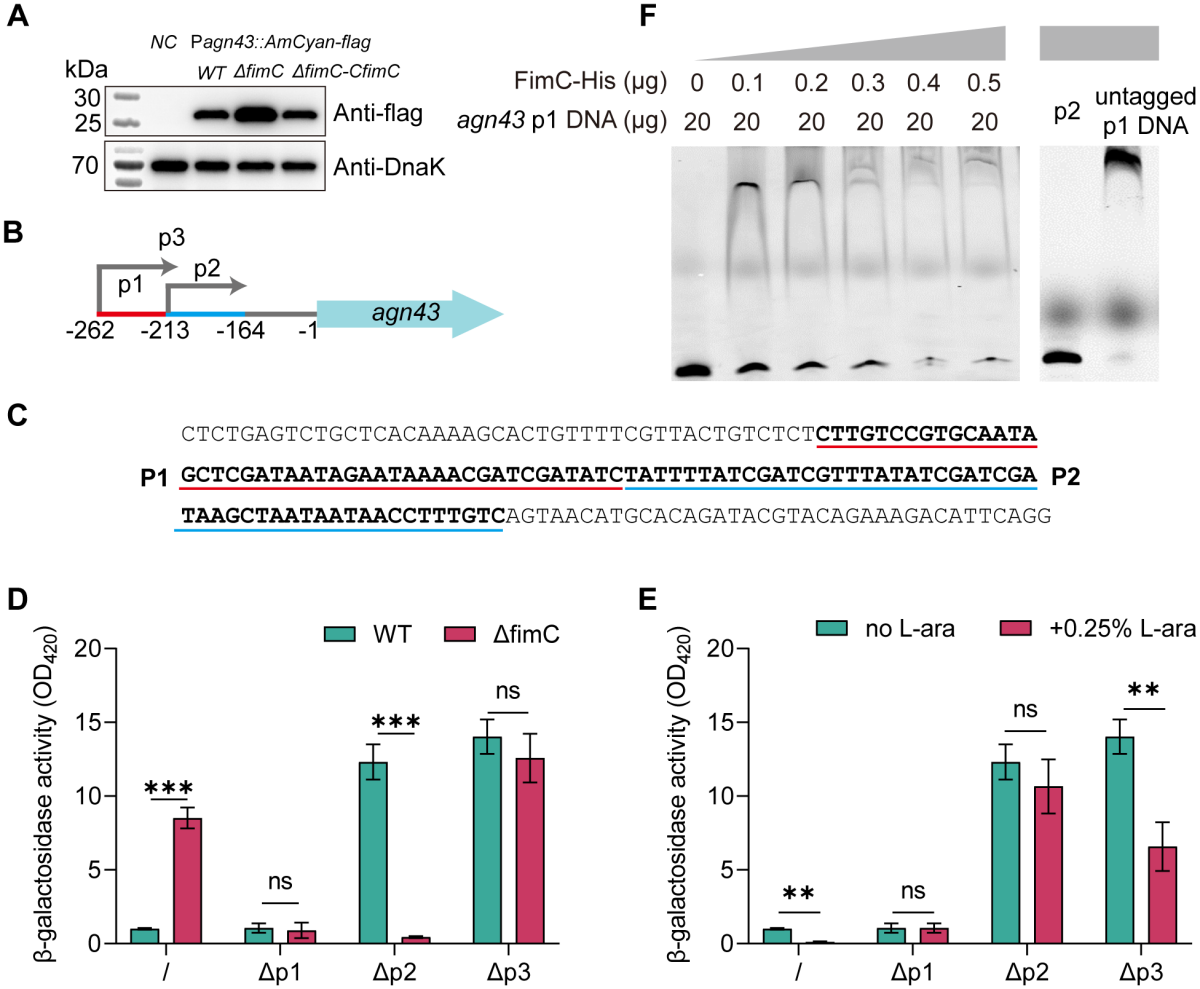
*agn43* promoter analysis and electrophoretic mobility shift assay (EMSA ) validation. **(A)** Western blot analysis of the *agn43* promoter activity. *agn43* was fused with flag-tagged AmCyan ORF and transformed to WT, Δ*fimC* or Δ*fimC*-C*fimC* strain. Samples were harvested and analysis by SDS-Page and Western blot. Anti-flag monoclonal antibody was used for analysis the expression of AmCyan. Anti-DnaK polyclonal antibody was used for reference (n=3). **(B)** Promoter patterns of *agn43*. **(C)** Sequence analysis of the *agn43* promoter region. Bold sequence indicated predicted promoters. **(D, E)** Different lengths of *agn43* promoters leads to changes of activity when responding to lacking or overexpression of *fimC* (Δp1 or Δp2 means the whole promoters lacking p1 or p2) (n=3) . **(F)** Validation of *fimC* binds to the *agn43* promoter region by EMSA.

Although the lack of *fimC* significantly increased the transcription of *agn43*, its regulatory function at the protein level remains unknown. To further confirm that the promoter activity of *agn43* was enhanced by the deletion of *fimC*, a plasmid that contained the *agn43* promoter and fused with the flag-tagged *AmCyan* ORF was constructed. Using Western blot, it was shown that the lack of *fimC* increased the expression of the flag-tagged AmCyan (**Figure 4A**).

To further confirm the regulatory relationship between *fimC* and *agn43*, a pBAD plasmid for the overexpression of *fimC* was used to detect the *agn43* promoter activity. Before detection, it was noted that the *agn43* promoter is predicted to have two regions—p1 (from −262 to −213 bp) and p2 (from −213 to −164 bp) (**Figure 4C**)—using an online promoter prediction tool (https://www.fruitfly.org/seq_tools/promoter.html). The promoter patterns are illustrated in **Figure 4B**. The promoters were then fused into a plasmid containing the *lacZ* reporter gene through detection of β-galactosidase (LacZ) activity. The aim was to establish that p1 or p2 is the primary promoter of *agn43*. Through the deletion of p1 or p2, the activity of the promoter was verified to determine whether the activity of p1 or p2 is affected. The results showed that the lack of p3 (from −262 to −164 bp) completely inhibited the promoter activity (**Figure 4D**). The same results were shown with the lack of p1. However, the lack of p2 showed significant difference compared with WT (*p* < 0.01) (**Figure 4D**), indicating that p1, and not p2, is the main promoter for the transcription of the *agn43* gene. By overexpression of *fimC*, it showed that the activity of Δp1 or p2 showed not significantly changed (*p* > 0.05), while a significant difference was found in p3 (*p* < 0.01) (**Figure 4E**). Taken together, these results showed that promoter p1 is crucial for the transcription of *agn43* and that *fimC* inhibits the transcription of *agn43* by inhibiting the promoter activity of p1.

### 3.6 *fimC* binds to the promoter region of *agn43*

It has been shown that *fimC* regulates the promoter activity and represses the transcription of *agn43*. Therefore, we speculate that *fimC* binds to the p1 promoter region of *agn43*. As the EMSA results showed, with increasing concentration of the *fimC* protein, the shift bands increased, while the free DNA decreased (**Figure 4D**). However, the p2 promoter DNA was used as a control, which showed no shift bands with increasing concentrations of *fimC*. In addition, 200 μM (~100-fold) p1 DNA without the cy5.5 tag was used as the negative control, which showed no shift bands. Taken together, *fimC* binds to the p1 promoter region of *agn43*.

### 3.7 *fimC* and *agn43* affect motility and the early stage of biofilm formation

**Figure 5:**
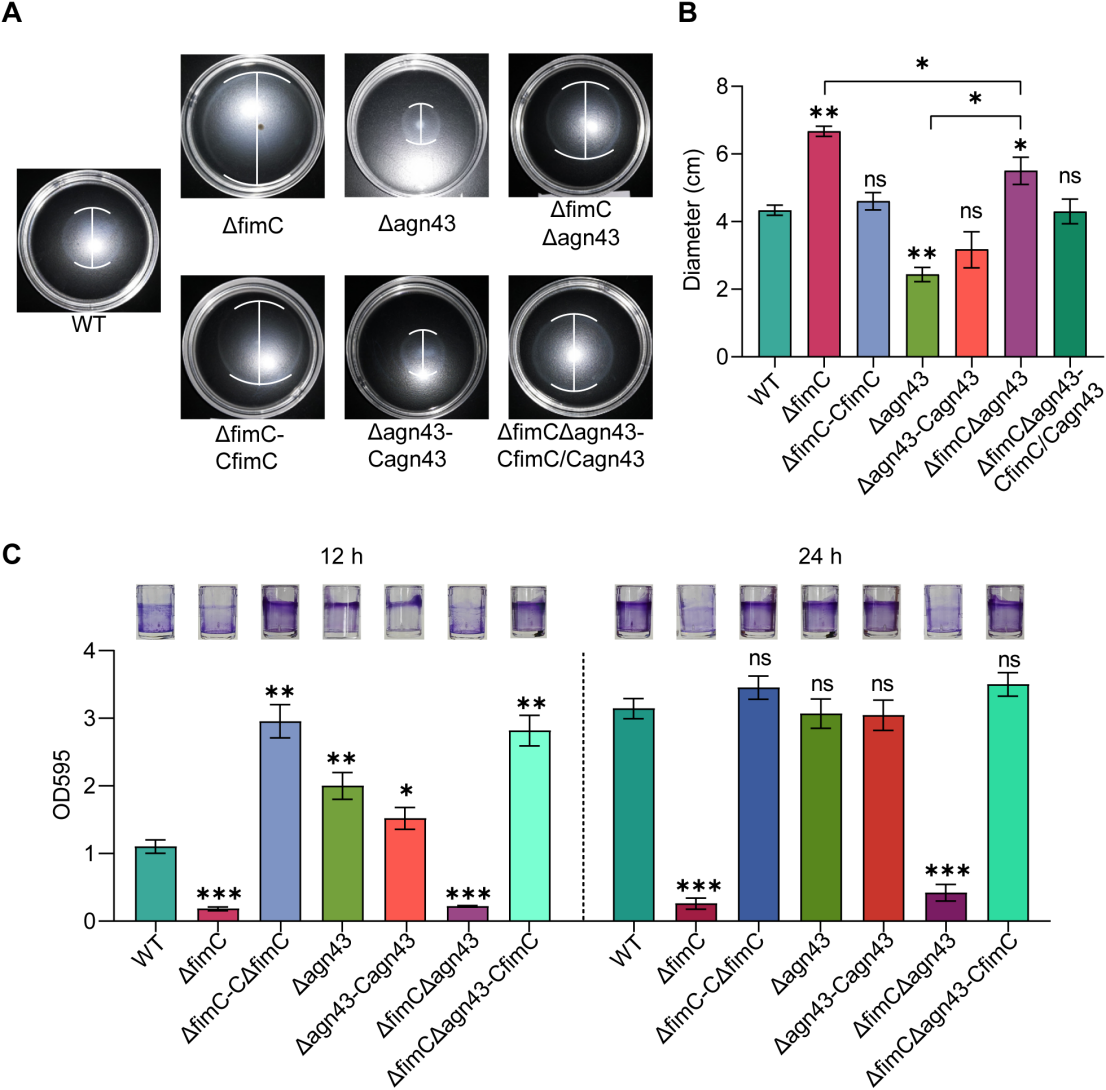
Detection of the motility and biofilm formation ability of *fimC* and *agn43* mutant strains. **(A, B)** Detection of the motility ability of *fimC* and *agn43* mutant strains (n=3). **(C)** Detection of the biofilm formation ability of *fimC* and *agn43* mutant strains (n=3).

As the transcriptomic data demonstrated the enrichment of the flagellum-mediated motility-related genes in the Δ*fimC* strains (**Figure 3B**), the flagellum-mediated motility of Δ*fimC*, Δ*agn43*, and Δ*fimC*Δ*agn43* was therefore determined. As shown, Δ*fimC* exerted significantly increased motility compared with the WT strain (*p* < 0.001) (**Figure 5A**), while the lack of *agn43* significantly decreased the motility (*p* < 0.01). In the Δ*fimC*Δ*agn43* strain, the motility was significantly decreased compared with that in Δ*fimC*, but it still showed high motility compared with Δ*agn43* (*p* < 0.01). These results indicate that *fimC* and *agn43* affect bacterial motility. However, it cannot be elaborated whether the regulation of motility by *fimC* is mediated by *agn43*.

Biofilm formation is an important biological characteristic regulated by several factors, such as adhesion, autoaggregation, motility, and c-di-GMP, among others. In order to explore whether the regulation of *fimC* and *agn43* on biofilm formation is related, the biofilm formation ability was examined. The results showed that, in the earlier (12 h) or the later stage (24 h) of biofilm formation, the deletion of *fimC* led to complete loss of the ability to form a biofilm (*p* < 0.001) (**Figure 5B**). However, the lack of *agn43* significantly increased the biofilm formation at 12 h (*p* < 0.01), but showed no significant difference at 24 h (*p* > 0.05) compared with the WT strain. In addition, the Δ*fimC*Δ*agn43* strain demonstrated complete inability to form a biofilm at either 12 or 24 h. In conclusion, these results indicate that *fimC* and *agn43* affect bacterial motility and biofilm formation and that, more importantly, *agn43* mediates the early stage of biofilm formation.

### 3.8 *fimC* and *agn43* coordinate to increase the adhesion of APEC to HD11 cells

**Figure 6:**
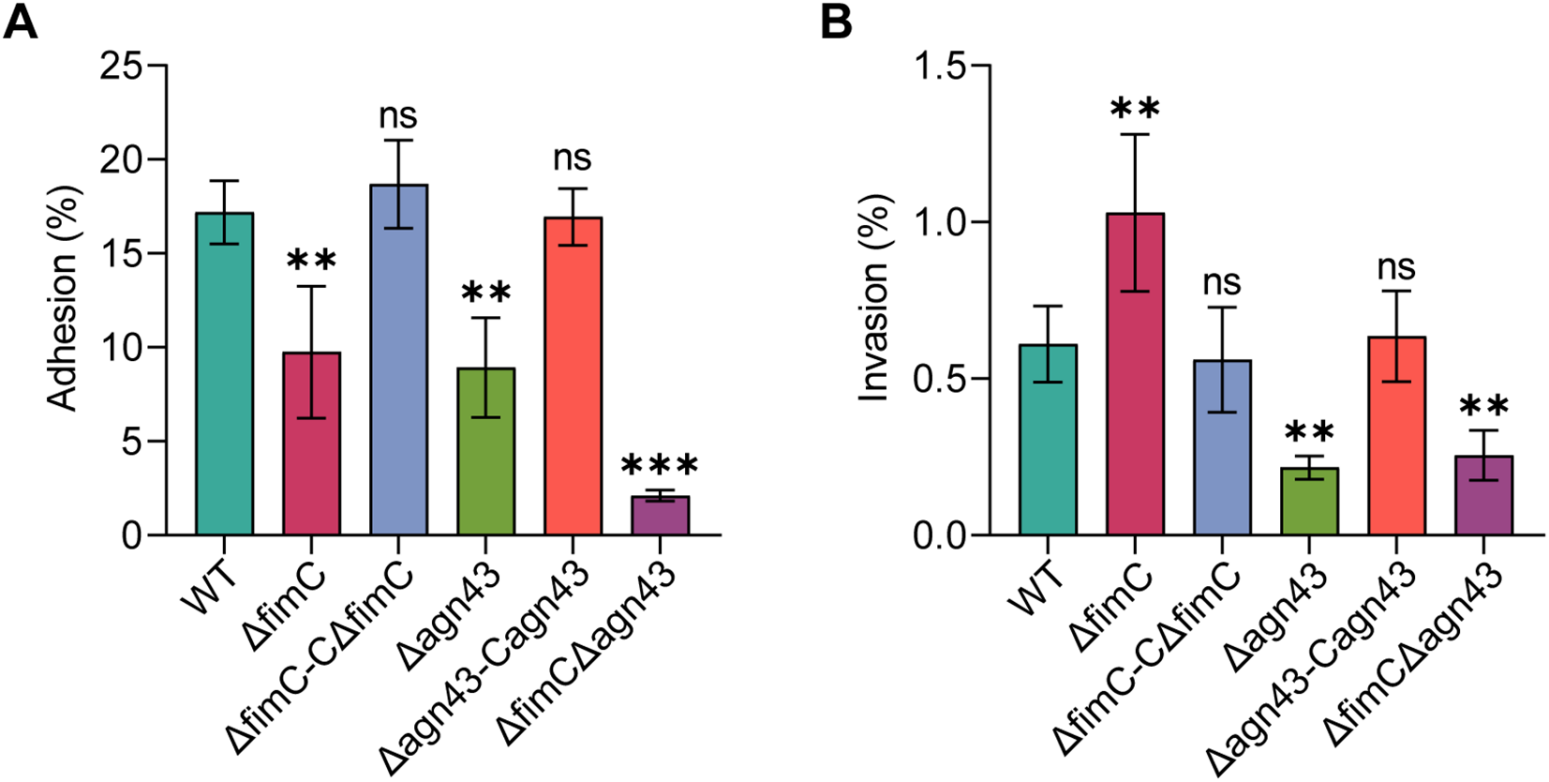
Detection of the adhesion and invasion ability of *fimC* and *agn43* mutant strains. **(A)** Detection of the adhesion ability of *fimC* and *agn43* mutant strains (n=3). **(B)** Detection of the invasion ability of *fimC* and *agn43* mutant strains (n=3) .

Adhesion and invasion are pathogenic processes mediated by bacterial fimbrial proteins such as type 1 fimbriae and other adhesins. To determine whether *agn43* is regulated by *fimC* during the adhesion to and invasion of avian macrophage HD-11 cells, the adhesion and invasion abilities were determined. The results showed that the lack of *fimC* significantly decreased the adhesion ability of APEC to HD-11 cells (*p* < 0.001). The Δ*agn43* strain also exhibited a significantly reduced adhesion ability to HD-11 cells compared with the WT (*p* < 0.01) (**Figure 6A**). Furthermore, the adhesion of Δ*fimC*Δ*agn43* was significantly decreased compared with either the Δ*fimC* or the Δ*agn43* strain (*p* < 0.01). These findings suggest that *fimC* and *agn43* synergistically enhance the adhesion of APEC to HD11 cells.

For the invasion ability, the results showed that the lack of *fimC* significantly increased the invasion ability (*p* < 0.01) (**Figure 6B**), while the deletion of *agn43* significantly decreased the invasion ability (*p* < 0.01). However, in Δ*fimC*Δ*agn43*, the invasion ability was significantly decreased and showed no difference compared with Δ*agn43* (*p* > 0.05). Taken together, these findings demonstrate that *fimC* and *agn43* coordinate to increase the adhesion of APEC to HD11 cells, but that *fimC* may suppress the invasive ability by negatively modulating *agn43*.

## 4 Discussion

Gram-negative pathogens utilize adhesins, filamentous protein complexes anchored to the outer membrane, known as fimbriae, to bind to the surface glycans of host cells and initiate infection (Hospenthal and Waksman, 2019; Jones et al., 1995; Lindberg et al., 1987; Martinez et al., 2000; Roberts et al., 1994). Type 1 fimbriae and the related P pili are among the most well-characterized pilus systems found in *E. coli* (Werneburg and Thanassi, 2018).

*In vivo*, the assembly of type 1 fimbriae follows the chaperone–usher pathway (Crespo et al., 2012; Werneburg and Thanassi, 2018) and relies on the periplasmic chaperone *fimC*. The *fimC* chaperone selectively recognizes the disulfide forms of the unfolded subunits and catalyzes their folding (Choudhury et al., 1999; Vetsch et al., 2004).

Aggregation is considered an important characteristic for bacterial survival during host infection (Klemm et al., 2006; Charbonneau and Mourez, 2008). Evidence accumulated in the literature suggests that *fimC* regulates autoaggregation. As expected, it was observed that the lack of *fimC* significantly increased the autoaggregation of APEC (**Figure 2A**). In addition, initially, it was believed that the deletion of *fimC* resulted in failure to synthesize the fimbriae, which further led to autoaggregation. However, the ΔfimA or the ΔfimD strain was completely unable to form fimbriae while having no effect on autoaggregation (**Figure 2A**). This provided proof that *fimC*, but not fimbriae, is crucial for the regulation of aggregation.

With further evidence, we showed the DNA-binding ability of *fimC*. As an atypical DNA-binding protein, *fimC*, which codes the PapD_N-PapD_C domain, did not bear any DNA-binding domain. The PapD_N or the PapD_C domain is a pilus and flagellar assembly chaperone for pilus and flagellar assembly. In all native *fimC*–subunit complexes, the periplasmic chaperone *fimC* significantly accelerates the folding of pilus subunits into a defined tertiary structure, increasing the process up to ~104-fold (Choudhury et al., 1999; Vetsch et al., 2004). These are notably faster chaperonin-bound subunits in archaic and alternative chaperone–usher pilus systems, where the subunits may only be partially folded (Pakharukova et al., 2018). This demonstrates the importance of *fimC* for type 1 fimbrial synthesis, as well as bacteria. Although there is no typical DNA-binding domain in *fimC*, such as the helix–turn–helix (HTH) DNA-binding domain that is widely spread in prokaryotic transcription regulatory proteins, we speculate that *fimC* may only bind to the promoter DNA and occupy the binding sites, leading to loss of the transcription regulatory modulation by other DNA-binding proteins. However, the premise of this hypothesis is that *fimC* has a higher affinity with DNA compared with the processes of typical DNA-binding domain proteins. However, in this study, although the binding and regulatory effects of *fimC* on the *agn43* promoter have been proven, the regulation of other genes remains unknown. Although the transcription levels of several genes were altered after *fimC* deletion (**Figure 3A**), these changes may not be directly caused by *fimC*.

In addition, it has been proven that the aggregation mediated by *agn43* was directly regulated by *fimC*. However, there was no evidence illustrating that the effects on motility, biofilm formation, and adhesion and invasion were directly regulated by *fimC* (**Figures 5A, B**). Although *fimC* and *agn43* have antagonistic effects on the regulation of motility and synergistic effects on the regulation of adhesion, *fimC* or *agn43* plays a regulatory role in the later stage of biofilm formation and invasion (**Figures 5C**, **6B**), forming the complex regulatory relationship and making it difficult to elucidate the regulatory network. The effects on the biological characteristic regulation between *fimC* and *agn43* need to be explored further.

It is worth noting that the flagellum-mediated motility and the c-di-GMP metabolism were enriched in transcriptomics, and both have significant regulatory effects on biofilm formation. c-di-GMP, as a ubiquitous secondary messenger in bacteria, affects multiple biological characteristics including biofilm formation and motility. However, due to the complexity of the regulatory network of c-d-GMP, with ~30 genes involved in c-di-GMP metabolism (Povolotsky and Hengge, 2016), it is difficult to establish whether there is a regulatory relationship between *fimC* and c-di-GMP. Nevertheless, there is still a potential that *fimC* could affect the biological characteristics of bacteria such as autoagglutination, biofilm formation, and motility by regulating the transcription of the c-di-GMP metabolic genes.

**Figure 7:**
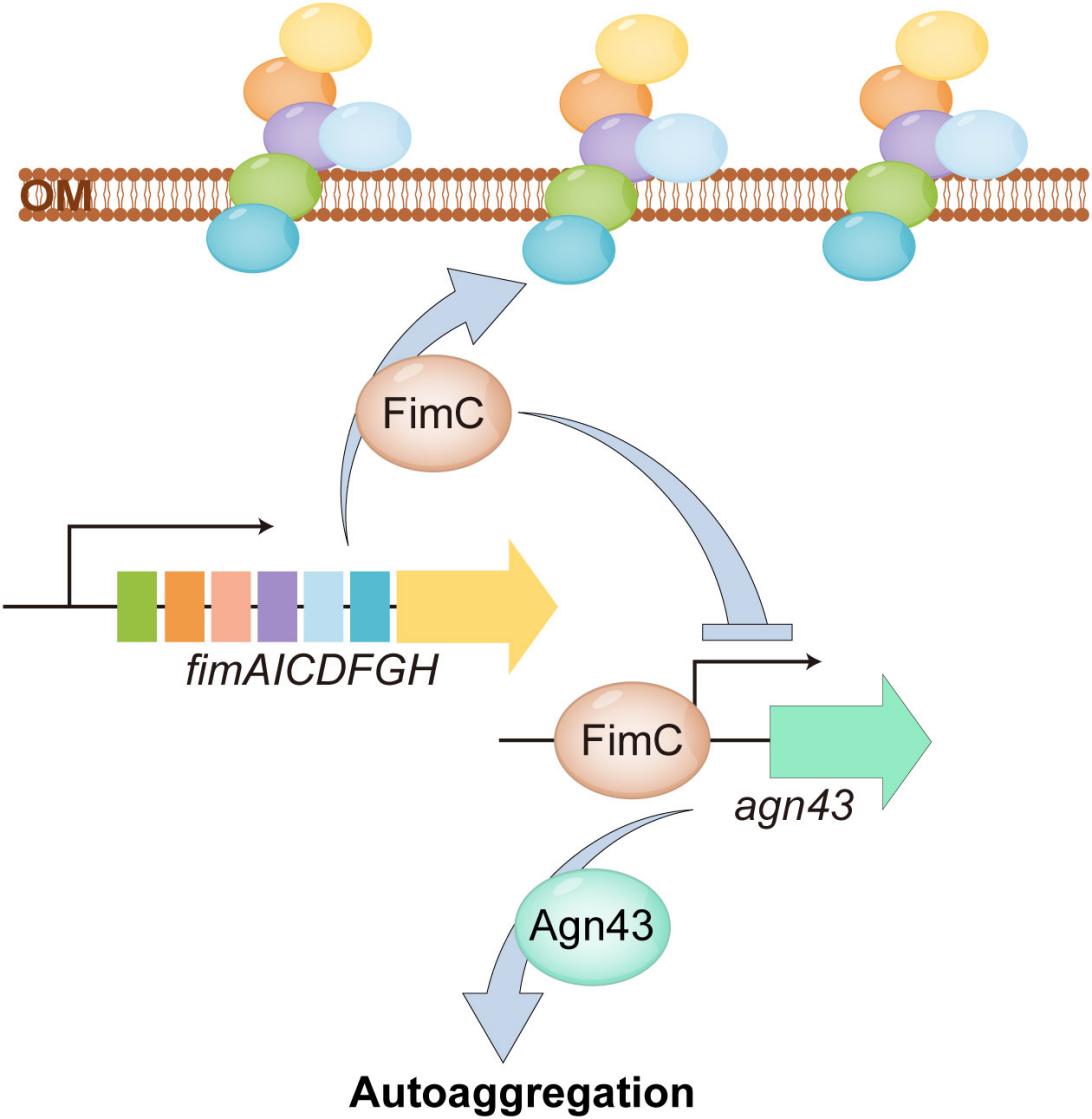
A diagram for illustrate the regulatory function of *fimC*. *fimC* as a chaperone protein mediates type 1 fimbrial synthesis. Meanwihle, *fimC* inhibits transcription of *agn43* by binding to its promoter regin, thereby mediating bacterial autoaggregation.

In conclusion, in this study, we demonstrated the regulatory relationship of *fimC* and *agn43* and confirmed that the effect of the lack of *fimC*, but not fimbriae, on autoaggregation is mediated by *agn43* (**Figure 7**). Both *fimC* and *agn43* have regulatory effects on biofilm formation, motility, and adhesion and invasion; however, the specific mechanism still needs to be explored further. This study provides new insights into the prevention and control of APEC and a novel idea for the development of fimbrial-based vaccines.

## 5 Conclusion

*fimC* is an essential component for the synthesis of type 1 fimbriae. However, in this study, it was demonstrated that *fimC* not only regulated fimbrial synthesis but also affected bacterial autoaggregation by regulating *agn43*. The results showed that *fimC*, rather than fimbriae, affected bacterial autoaggregation. Further investigation showed that *fimC* inhibited the transcription of *agn43* by directly binding to its promoter region. Finally, the effects of *fimC* and *agn43* on biofilm formation, motility, and the adhesion and invasion ability for HD-11 cells were also demonstrated. In conclusion, this study broadens the regulatory role of proteins without a DNA-binding domain on gene transcription, providing a new perspective for further understanding the function of *fimC*.

## Data Availability

The original contributions presented in the study are included in the article/**Supplementary Material**. Further inquiries can be directed to the corresponding authors.
