# Supplementary figures and images for: FimC binds to the promoter region of agn43 to modulate autoaggregation
Source: Front Cell Infect Microbiol. 2025 May 30;15:1591206. doi: 10.3389/fcimb.2025.1591206 (PMC12162486; doi:10.3389/fcimb.2025.1591206)

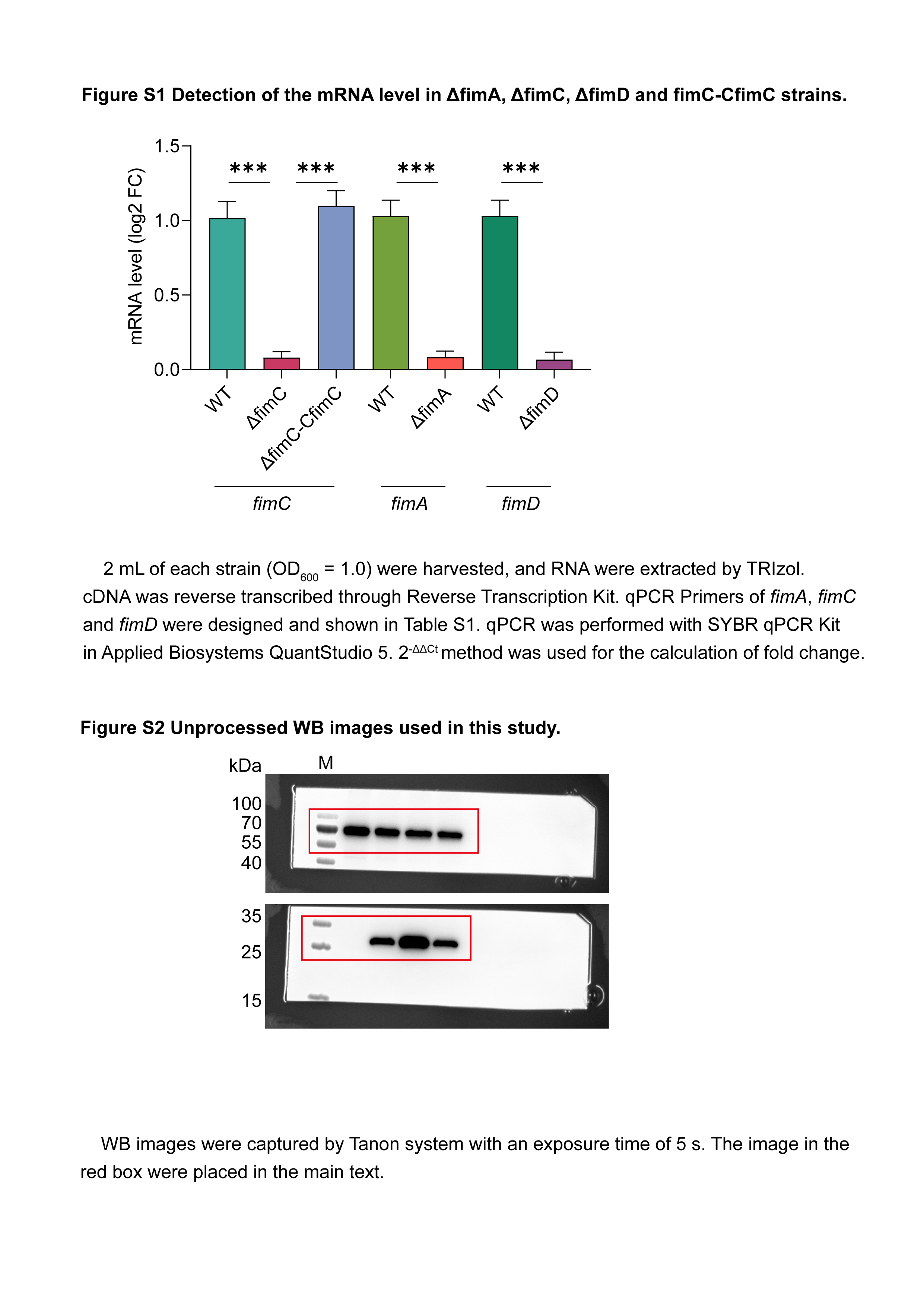

Supplement: Supplementary file 1 [file Image1.tif]
