# Supplementary material for: FimC binds to the promoter region of agn43 to modulate autoaggregation
Source: Front Cell Infect Microbiol. 2025 May 30;15:1591206. doi: 10.3389/fcimb.2025.1591206 (PMC12162486; doi:10.3389/fcimb.2025.1591206)
Supplement: Supplementary file 2 [file Table1.docx]

| **Strains or Plasmid** | **Genotype and/or characteristicsa** | **Source** |
| --- | --- | --- |
| **Strains** |  |  |
| APEC81 | Clinical isolated wild type APEC O78 serotype strain | Preserved in lab |
| ΔfimC | *fimC* gene knockout mutant strain based on WT | This study |
| ΔfimA | *fimA* gene knockout mutant strain based on WT | This study |
| ΔfimD | *fimD* gene knockout mutant strain based on WT | This study |
| Δagn43 | *agn43* gene knockout mutant strain based on WT | This study |
| ΔfimCΔagn43 | *agn43* gene knockout mutant strain based on ΔfimC | This study |
| ΔfimC-CΔfimC | Complemented strain of *fimC* based on ΔfimC | This study |
| Δagn43-Cagn43 | Complemented strain of *agn43* based on Δagn43 | This study |
| ΔfimCΔagn43-CfimC/Cagn43 | Complemented strainof *fimC* and *agn43* based on ΔfimCΔagn43 | This study |
| ΔlacZ | *lacZ* gene knockout mutant strain based on WT |  |
| ΔlacZ (p1::lacZ ) | Promoter of agn43 (p1) fused with *lacZ* gene based on ΔlacZ | This study |
| ΔlacZΔfimC (p1::lacZ ) | Promoter of agn43 (p1) fused with *lacZ* gene based on ΔlacZΔfimC | This study |
| ΔlacZ (p2::lacZ) | Promoter of agn43 (p2) fused with *lacZ* gene based on ΔlacZ | This study |
| ΔlacZΔfimC (p2::lacZ ) | Promoter of agn43 (p2) fused with *lacZ* gene based on ΔlacZΔfimC | This study |
| ΔlacZ (p3::lacZ ) | Promoter of agn43 (p3) fused with *lacZ* gene based on ΔlacZ | This study |
| ΔlacZΔfimC (p3::lacZ ) | Promoter of agn43 (p3) fused with *lacZ* gene based on ΔlacZΔfimC | This study |
| Δp1 | Deleted of promoter p1 of *agn43* gene based on WT | This study |
| ΔfimCΔp1 | Deleted of promoter p1 of *agn43* gene based on ΔfimC | This study |
| Δp2 | Deleted of promoter p2 of *agn43* gene based on WT | This study |
| ΔfimCΔp2 | Deleted of promoter p2 of *agn43* gene based on ΔfimC | This study |
| Δp3 | Deleted of promoter p3 of *agn43* gene based on WT | This study |
| ΔfimCΔp3 | Deleted of promoter p3 of *agn43* gene based on ΔfimC | This study |
| Δp1-pBAD-fimC | Overexpressing of *fimC* based on Δp1 | This study |
| Δp2-pBAD-fimC | Overexpressing of *fimC* based on Δp2 | This study |
| Δp3-pBAD-fimC | Overexpressing of *fimC* based on Δp3 | This study |
| **Plasmids** |  |  |
| pSTV28-fimC | Low-copy plasmid for expressing of *fimC* in APEC | This study |
| pTWV228-agn43 | Low-copy plasmid for expressing of *agn43* in APEC | This study |
| pET28a-fimC | High-copy plasmid for expressing *fimC* in BL21(DE3) | This study |
| pKD46 | Phage λ-derived Red recombination system for gene knockout | Preserved in lab |
| pKD3 | Chloramphenicol resistance (Cm^R^) ORF for gene knockout | Preserved in lab |
| pCP20 | Phage λ-derived Red recombination system for gene knockout | Preserved in lab |
| pBAD-fimC | High-copy plasmid for expressing *fimC* in APEC | Preserved in lab |

TABLE S1 Strains and plasmids used in this study.
