# Supplementary material for: FimC binds to the promoter region of agn43 to modulate autoaggregation
Source: Front Cell Infect Microbiol. 2025 May 30;15:1591206. doi: 10.3389/fcimb.2025.1591206 (PMC12162486; doi:10.3389/fcimb.2025.1591206)
Supplement: Supplementary file 3 [file Table2.docx]

TABLE S2 Primers used in this study.

| **Primers** | **Sequence (5′-3′)** | **Target gene** |
| --- | --- | --- |
| **Primers for**  **gene knockout** |  |  |
| cm-fimC-F | GATAATGTGATAACAGGAACAGGACAGTGAGTAATAAAAATGTAGGCTGGAGCTGCTT | *fimC* gene mutant strain construction and identification |
| cm-fimC-R | CGTTATTCCATTACGCCCGTCATTTTTGGGGTAAGCATATGAATATCCTCCTTAGTTC |  |
| fimC-in-F | TTTAATTCAATCATGGGTGG |  |
| fimC-in-R | ATTTTGATTTATCCATTGAC |  |
| fimC-out-F | GGCATCGCCAATGCACAGGC |  |
| fimC-out-R | TGTTTCGCTGGTAAAGTCTT |  |
| cm-agn43-F | GGTACCGGCTTTTTTATTCACCCTCTGTAAGGAAAAGCTTTGTAGGCTGGAGCTGCTT | *agn43* gene mutant strain construction and identification |
| cm-agn43-R | TGATGACCGGGGCCTGAAGGGGAGTGACCTGATTACATATGAATATCCTCCTTAG |  |
| agn43-in-F | GAAACGACATCTGAACACC |  |
| agn43-in-R | CACTCTCTGAAGACCACCA |  |
| agn43-out-F | GAATTGCACACAACGGACTG |  |
| agn43-out-R | GGATCGGGTAATAACTGGCC |  |
| **Primers for plasmid construction** |  |  |
| pSTV28- fimC-F | CGGGATCCGATAATGTGATAACAGGAAC | Expressing of *fimC* |
| pSTV28- fimC-R | CCAAGCTTCGTTATTCCATTACGCCCGT |  |
| pTWV228-agn43-F | CGGGATCCATGAAACGACATCTGAACAC | Expressing of *agn43* |
| pTWV228-agn43-R | CCAAGCTTTCAGAAAGTCACATTCAGTG |  |
| pBAD-fimC-F | CGGGATCCGATAATGTGATAACAGGAAC | Expressing of *fimC* |
| pBAD-fimC-R | CCAAGCTTCGTTATTCCATTACGCCCGT |  |
| pET28a-fimC-F | CGGGATCCGATAATGTGATAACAGGAAC | Expressing of *fimC* |
| pET28a-fimC-R | CCAAGCTTCGTTATTCCATTACGCCCGT |  |
| Primers for RT-PCR |  |  |
| RT-dnaE-F | TTTGACGCACACGAAATCCG | *dnaE* |
| RT-dnaE-R | TTGCTGCGGCGAATAGTTAC |  |
| RT-agn43-F | AGAAAACACGGGTGGATGAC | *agn43* |
| RT-agn43-R | GATGTCCGACAGTGGTGTTG |  |
| RT-fimA-F | TTGCGATACCAATGTTGCAT | *fimA* |
| RT-fimA-R | TCAGGGTTGTTTGCTCACTG |  |
| RT-fimB-F | GGTGGTAATGCTGGGTTGTC | *fimB* |
| RT-fimB-R | GCTCTATCCCAGATGCCGTA |  |
| RT-fimD-F | AGAATCGGATTGGGGGTAAC | *fimD* |
| RT-fimD-R | TCTCGCTCAAGCCAGGTATT |  |
| RT-fimE-F | CTAACTGGAAAGGCGCTGAC | *fimE* |
| RT-fimE-R | GATGCCCGAGATAATCCTGA |  |
| RT-fimF-F | CTCCTGTCGTTCCATTTCGT | *fimF* |
| RT-fimF-R | CGATGGAGCATTAAGGGGTA |  |
| RT-fimG-F | CATGATGTTGCGCTTGAGTT | *fimG* |
| RT-fimG-R | GTTTTGGTTGCGCCAGTATT |  |
| RT-fimH-F | TATTTGACGCCTGTGAGCAG | *fimH* |
| RT-fimH-R | AGGAATTGGCACTGAACCAG |  |
| RT-lsrR-F | TTTTGATGCAAAAGGTGACG | *lsrR* |
| RT-lsrR-R | ATAACCGCCTTTCATTGCAG |  |
| RT-lsrK-F | CAACCATCACCATGATCAGC | *lsrK* |
| RT-lsrK-R | AATGTGCCGGTTTCTTTGAC |  |
| RT-flhC-F | ACATAAGCTGCAGGCAAAGC | *flhC* |
| RT-flhC-R | TGCAACTTTCCAGCTGCAAC |  |
| RT-flhD-F | GCCGTAGCTATCGCAAAGC | *flhD* |
| RT-flhD-R | CCATACTTTCCCGCAGCTTG |  |
| RT-dgcE-F | CATTGAAGTGCCGTTGTTTG | *dgcE* |
| RT- dgcE -R | CCACCATGTTACGCAGTGTC |  |
| RT- pdeH -F | TCTGGATATCGAAGGGATCG | *pdeH* |
| RT- pdeH -R | AGGCGCAGAACTTCACTCTC |  |
| RT-csgA-F | TGACCTTGCCATAGCTTGTG | *csgA* |
| RT- csgA -R | GCCAAGGATATCGTCGAGAG |  |
| RT-csgD-F | TGGTAAACACTTGCCCCATA | *csgD* |
| RT- csgD-R | GATACCCAGGGTGACGATGT |  |
| RT-bcsA-F | GTGAGCCGATCCCTCTTGTA | *bcsA* |
| RT- bcsA -R | GTATCAGGTAGCGGCGGTAA |  |
| **Primers for EMSA** |  |  |
| p1-F | CGGAATTCACAGTCTGCCAGGGGAAATG | p1 promoter of *agn43* |
| p1-R | CCCATATGAAGCTTTTCCTTACAGAGGG |  |
| p2-F | GCTGCAGGGAAATGCCTGATTACATTG | p2 promoter of *agn43* |
| p2-R | GGGGTACCTAACCACTATAAACGATCGA |  |
| **Primers for promoter knockout** |  |  |
| p1–UF | GTACTGTGGCTGATTAAAGCCG | p1 promoter of *agn43* mutant strain construction and identification |
| p1–UR | CAGCCGTTAAAGGCATTTCCCCTGGCAGA |  |
| p1-CF | GGAAATGCCTTTAACGGCTGACATGGGAAT |  |
| p1-CR | AAAAAGCCGGGTGTAGGCTGGAGCTGCTTC |  |
| p1–DF | CAGCCTACACCCGGCTTTTTTATTCACCCTCT |  |
| p1–DR | TGCCAGCCCTTCTCATTAATGA |  |
| p1-in-F | ACATTGCGTTAACCAACTGA |  |
| p1-in-R | AAAAAGCCGGTACCCACAAA |  |
| p1-out-F | CGGAATCTGTCCGGTCTCAG |  |
| p1-out-R | TAAGAGACAGTGCAACCGCC |  |
| p2–UF | GGGACAGGGATGCAGAGTAT | p2 promoter of *agn43* mutant strain construction and identification |
| p2–UR | CAGCCGTTAAAGGCATTTCCCCTGGCAGA |  |
| p2-CF | GGAAATGCCTTTAACGGCTGACATGGGAAT |  |
| p2-CR | TAACCACTATGTGTAGGCTGGAGCTGCTTC |  |
| p2–DF | CAGCCTACACATAGTGGTTAATGGCCTGCG |  |
| p2–DR | GCGGTACCGCCATTTTGTAT |  |
| p2–OF | TACCGTGACATTCTGCCTGA |  |
| p2–OR | CAGCCGTTAAAGATTCAGCTGCCTTTTCGC |  |
| p2-inF | AGCTGAATCTTTAACGGCTGACATGGGAAT |  |
| p2-inR | AAAAAGCCGGGTGTAGGCTGGAGCTGCTTC |  |
